# Intimal Sarcoma after Endovascular Abdominal Aortic Aneurysm Repair

**DOI:** 10.3400/avd.cr.22-00057

**Published:** 2022-12-25

**Authors:** Masaki Komatsu, Kazuki Naito, Shuji Chino, Haruki Tanaka, Hajime Ichimura, Takateru Yamamoto, Ko Nakahara, Megumi Fuke, Yuko Wada, Tatsuichiro Seto

**Affiliations:** 1 Department of Cardiovascular Surgery, Shinshu University School of Medicine, Matsumoto, Nagano, Japan

**Keywords:** intimal sarcoma, endovascular aneurysm repair, stent graft infection

## Abstract

A 72-year-old man, who was treated 10 years earlier with endovascular aortic aneurysm repair, presented with a fever. Considering the concern of stent graft infection, the patient was treated with antibiotics, but his condition did not improve. He underwent stent graft resection and reconstruction with a Dacron graft. Pathological analysis of the aortic wall and computed tomography revealed recurrent intimal sarcoma, and the patient underwent resurgery. During follow-up, he underwent two additional resections for local recurrence, but he died 17 months later. Our results suggest that intimal sarcoma should be considered during the follow-up after endovascular aortic aneurysm repair.

## Introduction

Intimal sarcoma of the abdominal aorta is rare. Intimal sarcoma presents with intraluminal growth with obstruction of the lumen of the vessel of origin and seeding of emboli, and it is difficult to diagnose and is often diagnosed in surgical specimens or at autopsy.^1)^ Owing to early blood metastasis and resistance to chemotherapy, patients with intimal sarcoma have a poor prognosis. In this report, we describe our experience with intimal sarcoma following endovascular aortic aneurysm repair (EVAR). This case report has been approved by the institutional committee on the ethics review board (registration number: 5504).

## Case Report

**Figure 1:**
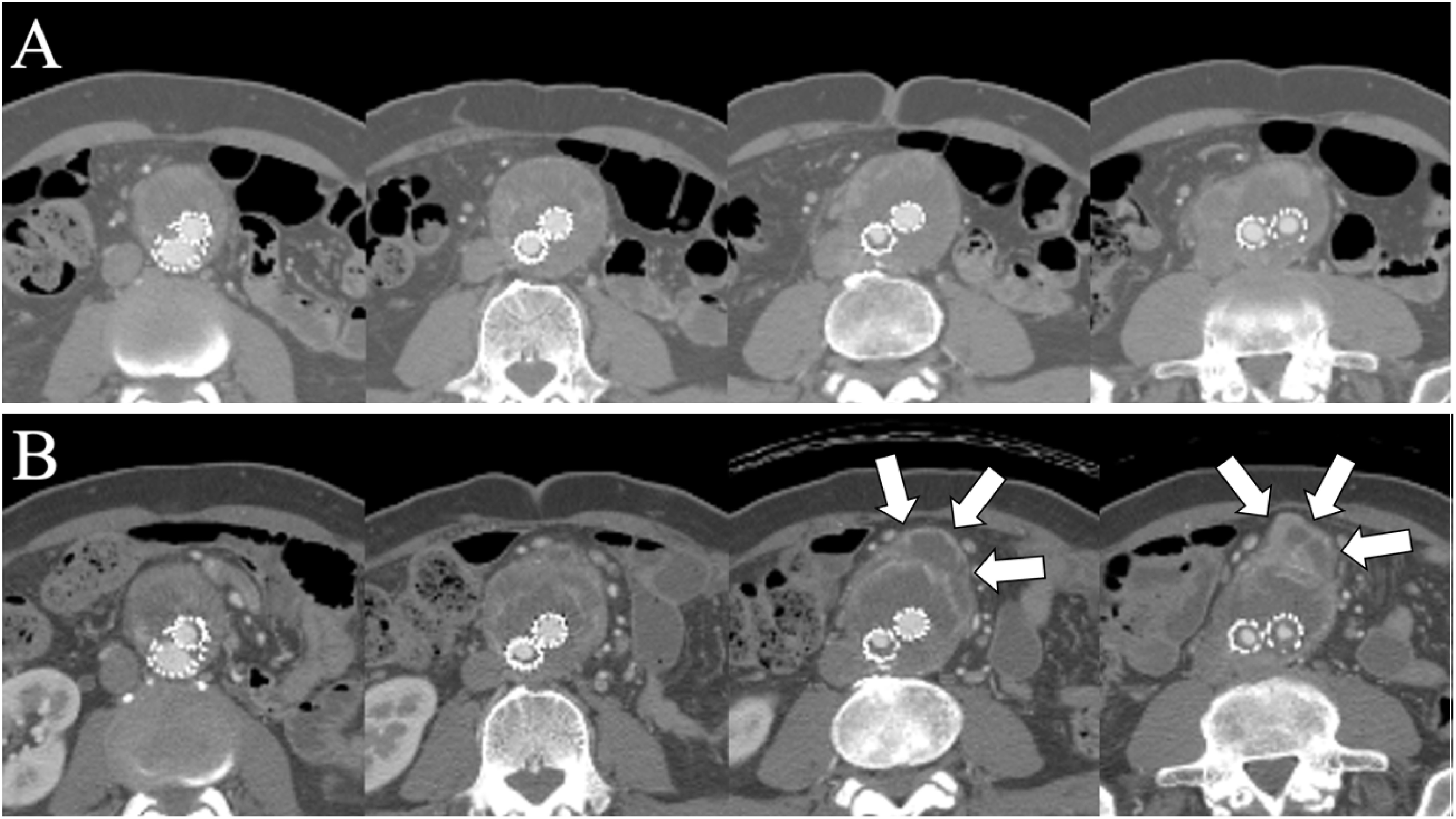
Fig. 1 Serial computed tomography (CT) image of aortic aneurysm follow-up. (**A**) CT surveillance scan four months before the patient became symptomatic. (**B**) CT scan at the time of presentation with an increase in aneurysm size from 4.8 to 5.5 cm, periaortic thickening, and periaortic inflammation (arrows). There was no endoleak during observation.

**Figure 2:**
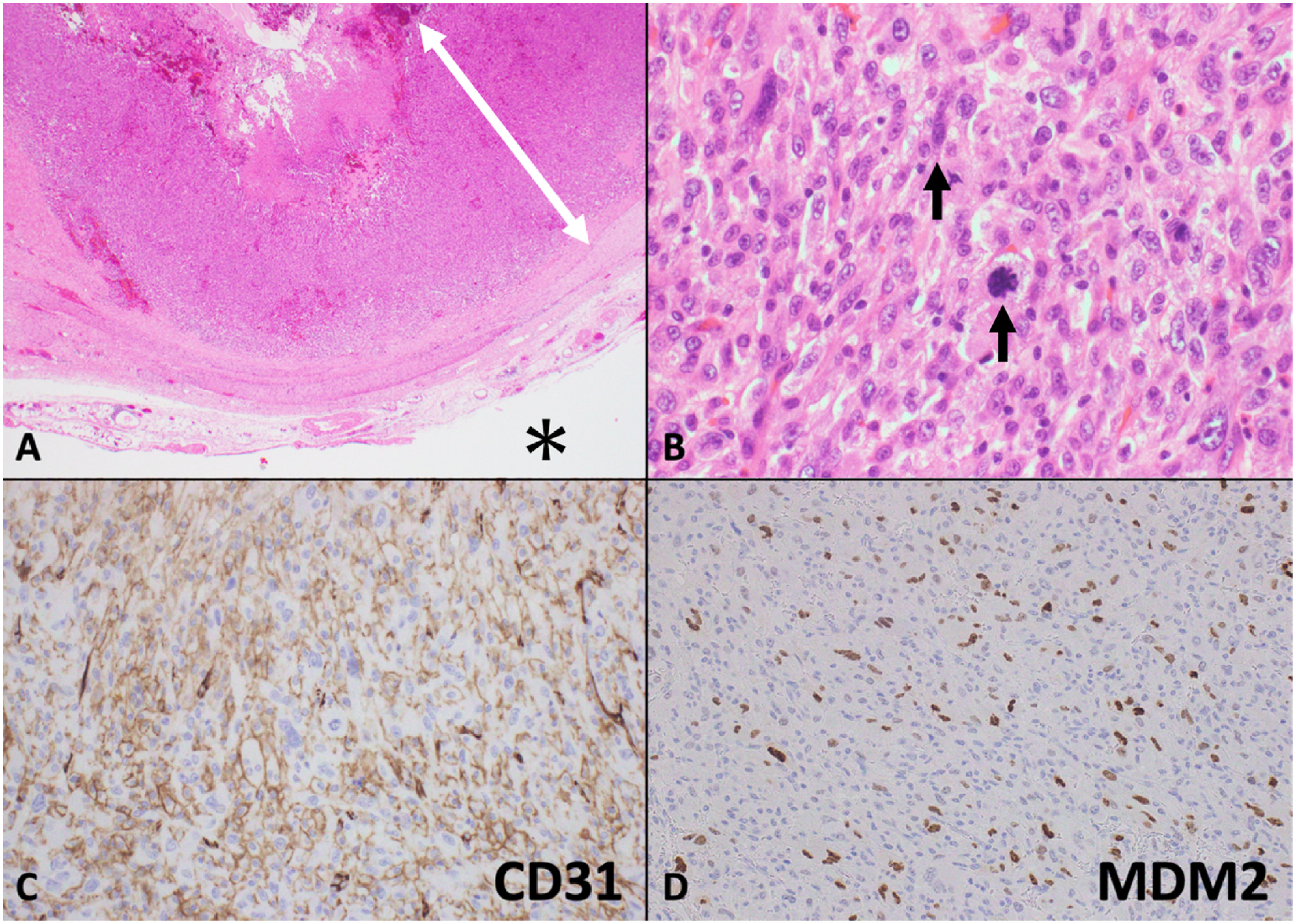
Fig. 2 Histology image of the vessel wall showed neoplastic lesions in the vascular intima (white arrow) and proliferation of spindle and pleomorphic cells (black arrows) (hematoxylin and eosin stain, ×20 [**A**] and ×400 [**B**]). * is outside of blood vessels. Immunohistochemical examination revealed strong positive staining for (**C**) CD31 and focal positive staining for (**D**) MDM2.

The patient was a 72-year-old man with a history of diabetes mellitus and EVAR for abdominal aortic aneurysm with a Zenith Flex stent graft (Cook Medical, Bloomington, IN, USA), which was placed at another institution 10 years before the visit to our clinic. He was admitted to the hospital of origin complaining of fever and physical weariness, which had persisted for four months previously. Symptoms did not improve after antibiotic treatment, and the patient was referred to our department. Laboratory tests at admission revealed an elevated C-reactive protein (10.6 mg/dL) and no leukocytosis (white blood cell count of 7480/mm^3^). Multiple sets of blood cultures were negative for bacteremia. Echocardiography showed a normal left ventricular ejection fraction and no valvular vegetation. Compared with the computed tomography (CT) scan from the previous hospitalization, enlargement of the aneurysm sac, periaortic thickening, and periaortic inflammation related to infection were noted in the present CT scan (**Fig. 1**). Evidence of irregular aneurysm sac dilatation and inflammatory changes was consistent with saccular mycotic aneurysms caused by stent graft (SG) infection. Considering the concern regarding SG infection, the patient underwent surgery. Significant periaortic inflammation with para-aortic lymphadenopathy was observed. The patient underwent SG resection and reconstruction with a rifampin-soaked bifurcated Dacron graft and aggressive local debridement of the aortic wall, including the adjacent lymph nodes. Intraoperative culture results were negative. After the Dacron graft was covered by the greater omentum, surgery was completed. The final histopathological examination of the surgical specimen revealed spindle and pleomorphic cells. Immunohistochemical examination revealed strong positive staining for CD31 and focal positive staining for MDM2 (**Fig. 2**). On the basis of these findings, we diagnosed the patient with intimal sarcoma. A postoperative CT scan showed tumors in the inferior vena cava, retroperitoneum, and colon, which were not observed in the preoperative CT scan. Fluorodeoxyglucose positron emission tomography/CT (FDG-PET/CT) scan showed pathologic tracer uptake in the inferior vena cava, retroperitoneum, and colon tumors revealed by CT, as well as in the iliac artery. On the basis of these findings, the patient was diagnosed with a recurrence of intimal sarcoma, and resurgery was performed one month after the surgery. We performed an additional resection of the bilateral iliac arteries with high FDG accumulation and graft replacement. Simultaneously, retroperitoneal lymph node dissection, ileocecal resection, inferior vena cava debridement, and reconstruction of the inferior vena cava with the bovine pericardium were performed. Postoperative chemotherapy was considered, but the patient did not wish to undergo chemotherapy and was discharged. Subsequently, the patient underwent resurgery twice for peritoneal recurrence, but he died 17 months after discharge.

## Discussion

Intimal sarcoma is a rare malignant neoplasm that arises in the aorta, and its defining features are intraluminal growth with obstruction of the lumen of the vessel of origin and seeding of emboli to peripheral organs.^1)^

Most patients with aortic intimal sarcoma are middle-aged, and the prognosis of intimal sarcomas is generally poor.^1)^ A PubMed literature search using the search terms EVAR and sarcoma revealed only 12 cases of primary abdominal aortic sarcoma after EVAR (**Table 1**).^2–9)^ The age of onset of aortic sarcoma after EVAR ranges from 56 to 85 years. Aortic sarcoma is defined as an intraluminal growth that obstructs the lumen of the vessel and emboli to the peripheral organs. However, the patients do not present with symptoms of embolism after EVAR, as in the present case. This investigation suggests that EVAR suppresses tumor growth in the aortic lumen and prevents embolism. However, most cases of aortic sarcoma after EVAR reported in the literature have described an association with foreign materials, such as Dacron or nickel and titanium alloys in SGs. In a study of 35 cases of angiosarcomas by Fenton et al., 23 cases were associated with the use of these prostheses.^4)^ Therefore, the physical characteristics of the implanted materials are considered essential factors for tumorigenesis. The duration from EVAR to tumorigenesis was 4–10 years, which also suggests an association with these prostheses.

**Table 1:** Table 1 Patient characteristics and treatment outcomes

Reference	Year	Age/Gender	Primary symptom	After EVAR	Metastatic lesion	Initial diagnosis	Definitive diagnosis of sarcoma	Treatment	Outcome
2	2012	85/M	General fatigue	7 years	Lumbar vertebra	Graft infection	Surgical specimens	Replaced	Dead In-hospital death
3	2012	56/M	Low back pain	6 years	—	Graft infection	Surgical specimens	Replaced	Dead 6 weeks
4	2014	73/M	Low back pain	7 years	Lumbar vertebra	Graft infection	Biopsy	Chemotherapy Radiation	Dead 24 months later
5	2016	60/M	Back pain, General fatigue	7 years	Peritoneal	Graft infection	Surgical specimens	Axi-BiF bypass	Dead In-hospital death
6	2016	69/M	Weight loss	8 years	—	Aneurysm enlargement	Biopsy	Chemotherapy Radiation	—
6	2016	77/M	Endoleak	—	Multiple	Aneurysm enlargement	Biopsy	—	Dead A few days later
6	2016	72/F	Low back pain	—	Multiple	Aneurysm enlargement	Biopsy	—	—
7	2017	81/M	Abdominal discomfort	5 years	Multiple	Aneurysm enlargement	Surgical specimens	—	Dead 2 days later
8	2019	68/M	Low back discomfort	4 years	Multiple	Graft infection	Surgical specimens	Replaced Chemotherapy	Alive
9	2019	78/M	Abdominal pain	10 years	Liver	Aneurysm enlargement	Surgical specimens	—	Dead A few months later
Our case	2021	72/M	Fever and Physical weariness	10 years	—	Graft infection	Surgical specimens	Replaced	Dead 17 months later

EVAR: endovascular aortic repair; M: male; F: female; Axi-BiF: axillo-bifemoral bypass

CT is a useful diagnostic tool for angiosarcomas. The imaging characteristics of aortic sarcoma include protrusive vegetation or nodular soft-tissue components, lack of atherosclerosis in the area of suspicion, heterogeneous thrombus, evidence of enhancement and neovascularity, and persistent enlargement of the excluded sac.^6)^ The CT findings of infected aneurysms include disruption of intimal calcification; irregular arterial lumen; and periaortic gas, fluid, and/or hematoma. However, CT findings of infected aneurysms with overlapping characteristics of intimal sarcoma are more common. Half of the case reports retrieved in this report were diagnosed with graft infection. The ultrasonographic findings of intimal sarcoma were reported by Kamran et al. to have a small mass-like component of intermediate echogenicity, which shows internal vascularity in the color Doppler image.^6)^ Conversely, the ultrasonographic findings of infected aneurysms are not characteristic, but the shape of the aneurysm is irregular, and the presence of air is noted. We have not confirmed any ultrasonographic findings in our case. PET scans show avid fluorodeoxyglucose uptake, but PET scans are not performed unless a tumor is suspected. It is important to be cautious of neoplastic lesions during follow-up after EVAR. However, imaging seldom permits the preoperative diagnosis of aortic sarcoma, and graft infection is often presumed, as in our case.^2–5,8)^ In previous studies,^2–9)^ preoperative diagnosis suspected graft infection in 6 of 11 cases, and in 7 cases, the diagnosis of aortic sarcoma was made by immunohistochemical examination of the surgical specimens. In our case, the final histopathological examination led to a diagnosis of intimal sarcoma. The treatment of intimal sarcomas remains difficult. Currently, in the absence of metastases, surgical resection of the tumor is the first-line treatment for intimal sarcoma. The role of adjuvant chemotherapy and radiotherapy remains unclear.^10)^ However, most patients with aortic sarcoma have metastasis at the time of diagnosis, due to which the five-year survival rate remains poor.

In a study published by Thalheimer et al., the median survival time was only 12.8 months after diagnosis, and metastases were present in 76.2% of cases.^10)^ However, there is a case report in which patients underwent en bloc resection of the aorta and common iliac vessels and survived for eight years from diagnosis to death.^10)^ Early diagnosis is important because complete resection of the primary lesion suggests the possibility of long-term survival. In this case, the patient was misdiagnosed with SG infection and underwent four months of antibiotic therapy, but the tumor expanded. During follow-up after EVAR, attention should be paid to neoplastic lesions, aneurysm sac enlargement, and graft infections.

## Conclusion

We presented a rare case of intimal sarcoma mimicking an infected EVAR. Intimal sarcomas after EVAR are rare and difficult to diagnose. Attention should be paid to neoplastic lesions, aneurysm sac enlargement, and graft infections during follow-up.
